# Using hyperspectral imagery to investigate large-scale seagrass cover and genus distribution in a temperate coast

**DOI:** 10.1038/s41598-021-83728-6

**Published:** 2021-02-18

**Authors:** Kenneth Clarke, Andrew Hennessy, Andrew McGrath, Robert Daly, Sam Gaylard, Alison Turner, James Cameron, Megan Lewis, Milena B. Fernandes

**Affiliations:** 1 School of Biological Sciences, The University of Adelaide, Adelaide, SA 5005 Australia; 2 Airborne Research Australia, PO Box 335, Salisbury South, SA 5106 Australia; 3 SA Water, GPO Box 1751, Adelaide, SA 5001 Australia; 4 South Australian Environment Protection Authority, GPO Box 2607, Adelaide, SA 5001 Australia; 5 Department for Environment and Water, GPO Box 1047, Adelaide, SA 5001 Australia; 6 College of Science and Engineering, Flinders University, GPO Box 2100, Adelaide, SA 5001 Australia

**Keywords:** Ecology, Environmental sciences

## Abstract

Seagrasses are regarded as indicators and first line of impact for anthropogenic activities affecting the coasts. The underlying mechanisms driving seagrass cover however have been mostly studied on small scales, making it difficult to establish the connection to seagrass dynamics in an impacted seascape. In this study, hyperspectral airborne imagery, trained from field surveys, was used to investigate broadscale seagrass cover and genus distribution along the coast of Adelaide, South Australia. Overall mapping accuracy was high for both seagrass cover (98%, Kappa = 0.93), and genus level classification (85%, Kappa = 0.76). Spectral separability allowed confident genus mapping in waters up to 10 m depth, revealing a 3.5 ratio between the cover of the dominant *Posidonia* and *Amphibolis*. The work identified the absence of *Amphibolis* in areas historically affected by anthropogenic discharges, which occasionally contained *Posidonia* and might be recovering. The results suggest hyperspectral imagery as a useful tool to investigate the interplay between seagrass cover and genus distribution at large spatial scales.

## Introduction

Seagrass meadows are hotspots for marine biodiversity, providing ecological functions that encompass biological, geochemical and physical needs of marine life^1^. As a transition zone to the open ocean, seagrasses further provide a variety of ecosystem services to humans. These include among others opportunities for recreation, protection from erosion and sea level rise, carbon sequestration, offsetting of ocean acidification, and breeding and nursery habitat for economically important species^2–4^. These functions and services are however threatened by the high sensitivity of seagrasses to water quality^5^, exacerbated by their generally high level of connectivity with neighboring habitats^6^. Successful management of these systems thus is intrinsically linked to a broader understanding of the dynamics of meadows at the scale of the seascape (100 s m^2^ to 1000 s of km^2^)^7^.

Remote sensing approaches provide an opportunity to inform management through integration of data at meaningful spatial scales, but mapping of subtidal seagrasses is hindered by many factors including water reflection, scattering and absorption of light^8^. Despite these limitations, aerial photography and satellite imagery have been used extensively to map change in seagrass meadows during the last century. Large worldwide losses have been documented as a result of urbanization and land-use change^9^, while the curbing of anthropogenic pressures has more recently seen a resurgence in regions as diverse as Chesapeake Bay, Southwest Florida estuaries, the Baltic Sea, Atlantic coasts of Europe, and the Mediterranean Sea^10–13^. A combination of ecological and remote sensing research indicates that complex mechanisms are associated with seagrass recovery, from small scale dispersal to large scale connectivity, patch recruitment, rhizome elongation and patch coalescence, of individual or multiple species^7^.

While at a broad geographical scale physical and chemical drivers define the dominant climax species in the recovery sequence, time since disruption and species succession play a critical role in determining which species are present at particular stages^14^. As seagrasses are lost, denuded areas become more energetic and early recruitment favours species with higher resistance to waves and currents, combined with high reproductive output^15^. These early colonisers act as ecosystem engineers and modify local conditions, allowing the establishment of other species, which in time come to dominate the system. The susceptibility of individual species to external drivers is thus an important determinant in the successional sequence and overall recovery time^16^. Changes in seagrass cover however are usually mapped as seagrass presence or absence, with less information available to determine mechanisms of change, such as the interplay between species, or the progression of cover density (e.g. from sparse to dense). Progress in remote sensing technology has provided a pathway to investigate these knowledge gaps, particularly through increased spatial and spectral resolution associated with the development of multispectral and hyperspectral sensors^17^.

In this study we used airborne hyperspectral imagery to investigate seagrass cover and genus distribution in a coastal metropolitan region (Adelaide, South Australia) dominated by *Posidonia* and *Amphibolis*, two temperate seagrass genera with distinct life history traits. *Posidonia* is considered a persistent genus highly resistant to disturbance^18^. Once lost, recovery is compromised by comparatively low and unreliable seed production and slow rhizome growth, among other factors. *Amphibolis* in contrast is considered an opportunistic genus, with the ability to release seedlings that can rapidly colonise areas where seagrass loss resulted in increased hydrodynamic forcing^19^. As the seeds of *Posidonia* lack dormancy and *Amphibolis* relies on the dispersal of seedlings, recovery is dependent on short windows of opportunity for the successful establishment of new recruits^20^. This study investigated the benefits and limitations of the use of airborne hyperspectral imagery to differentiate these seagrass genera in the seascape, and map overall seagrass cover. The data was then interpreted in terms of natural or anthropogenic drivers driving seagrass spatial dynamics.

## Results

**Figure 1:**
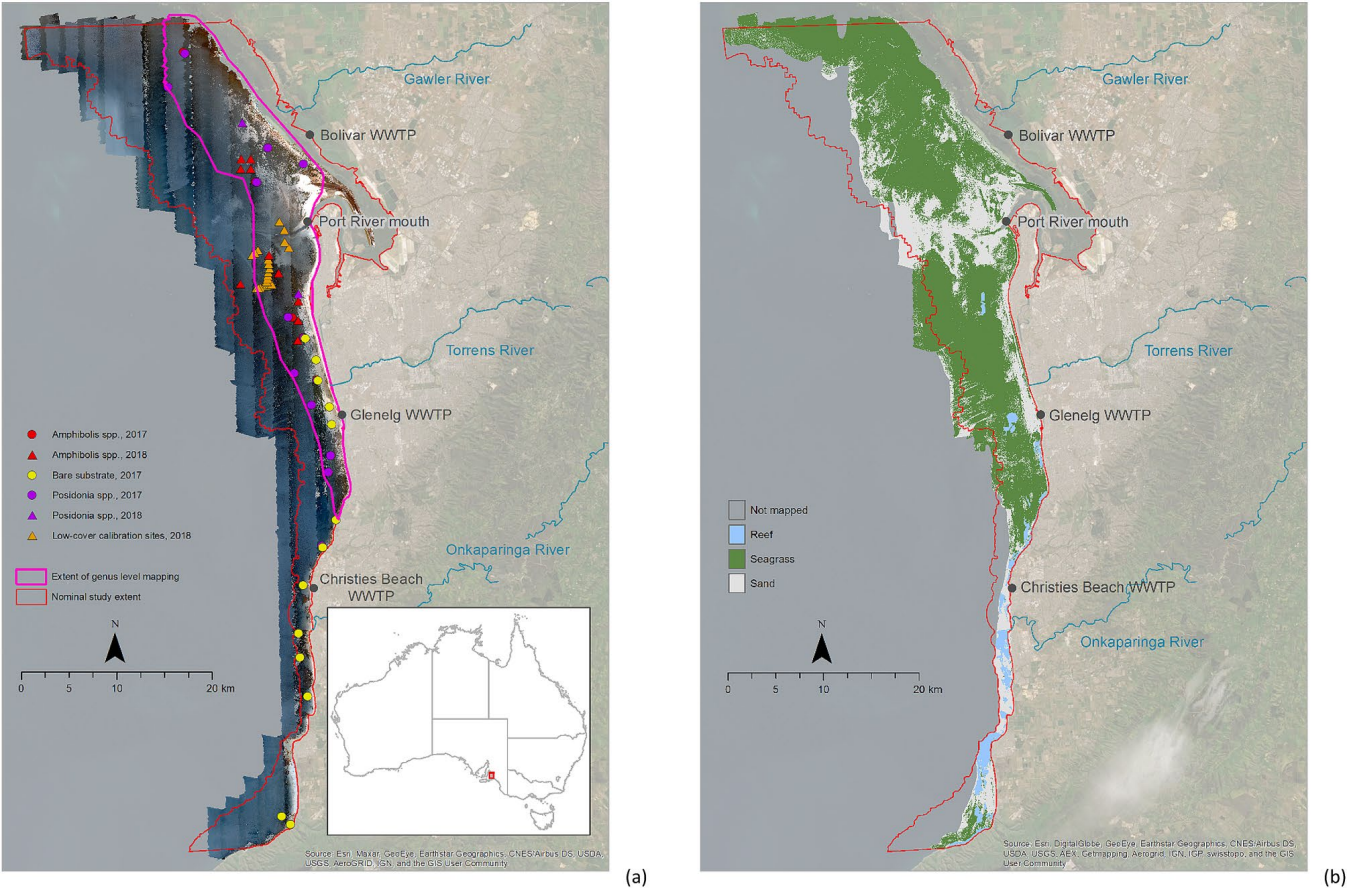
Sunglint corrected, pseudo true-colour image composite of the 2018 hyperspectral imagery (**a**), and benthic cover classification of this imagery (**b**). On the left panel, the nominal study area is outlined in red, and the extent of genus classification (Fig. 3) in purple. Field locations of large patches of homogeneous *Amphibolis*, *Posidonia* and sand cover used for accuracy assessment are differentiated by year of record (circle = 2017, triangle = 2018). Location of sites further investigated in 2018 to clarify suspected low cover are also presented. Map created in ESRI ArcGIS 10.7; terrestrial ESRI Basemap World Imagery (https://www.esri.com/en-us/arcgis/products/arcgis-enterprise).

The overall accuracy of benthic cover mapping was 98%, with only one sand site mapped as seagrass (Table 1). Although this accuracy assessment was based on a relatively small number of sites, a high Cohen’s kappa coefficient of 0.93 confirmed very strong agreement between field data and mapped cover. The classification revealed 42,298 ha of seagrass (70% of the study area) and 18,395 ha of sand (Fig. 1b). Sand was mostly prevalent in the vicinity of the Port River shipping channel, and in the first 1–2 km from shore north and south of the Torrens River. Visual fragmentation of seagrass cover also occurred further offshore south of the Torrens River and off the Gawler River.

**Table 1:** Confusion matrix based on number of sites, and accuracy for the benthic cover classification.

Mapped cover	Reference (field) data			User’s accuracy (%)
	Seagrass	Sand	Total	
Seagrass	**30**	**1**	31	97
Sand	**0**	**9**	9	100
Total	30	10	40	
Producer’s accuracy (%)	100	90		
Overall accuracy (%)	98

**Figure 2:**
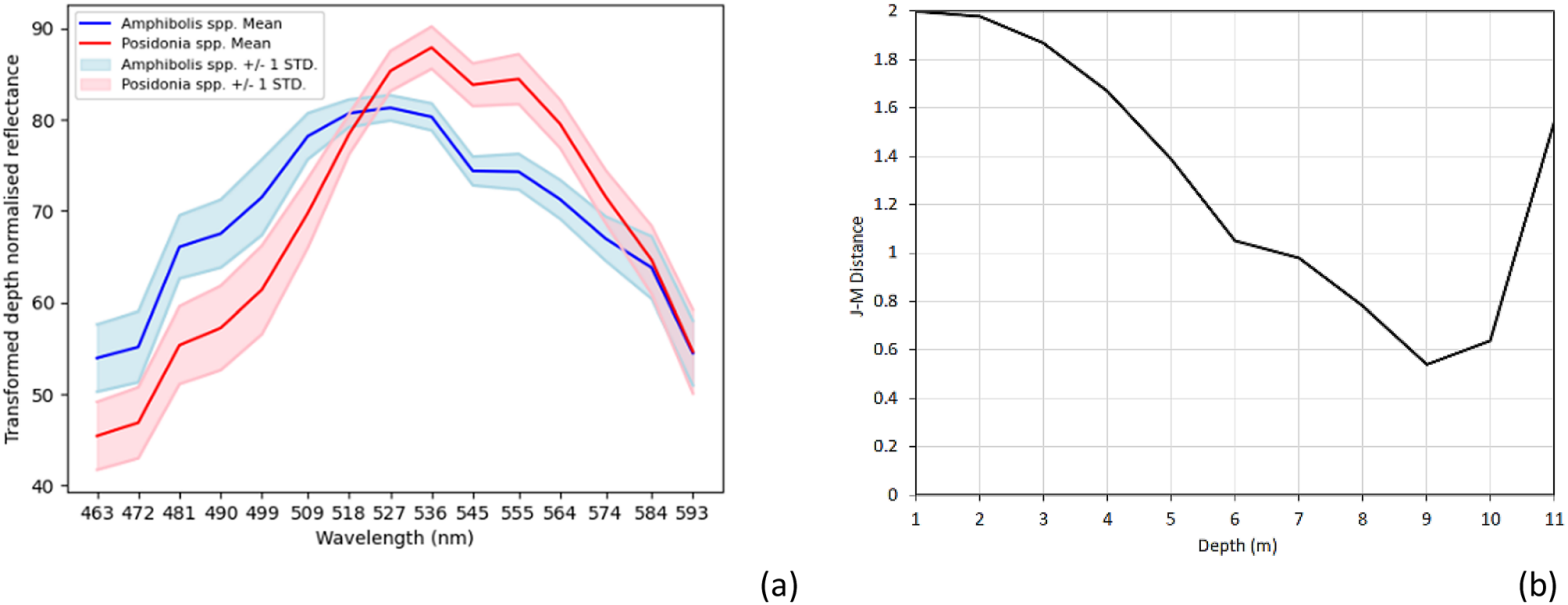
Mean spectral signature of *Amphibolis* and *Posidonia* in shallow (1–2 m) waters (**a**), and spectral separability indicated by Jeffries-Matusita (J-M) distance with water depth (**b**).

The analysis of genus spectral separability was performed on the highest quality portions of the imagery through the visual selection of reference sites, thus establishing separability under near ideal conditions. Jeffries-Matusita (J-M) distance followed an approximately linear decline with water depth, starting with perfect separability at 1–2 m and decreasing to a minimum at 9 m (Fig. 2). True separability probably continues to decline with depth, but an artefactual increase was calculated from 9 to 11 m due to the difficulty in locating reference sites in this depth range. The genus level classification was thus limited to 10 m water depth. The smaller area mapped to genus level (29,196 ha) in comparison to total benthic cover (60,693 ha) (Fig. 1a) highlights this sensitivity of the genus classification to water depth.

**Figure 3:**
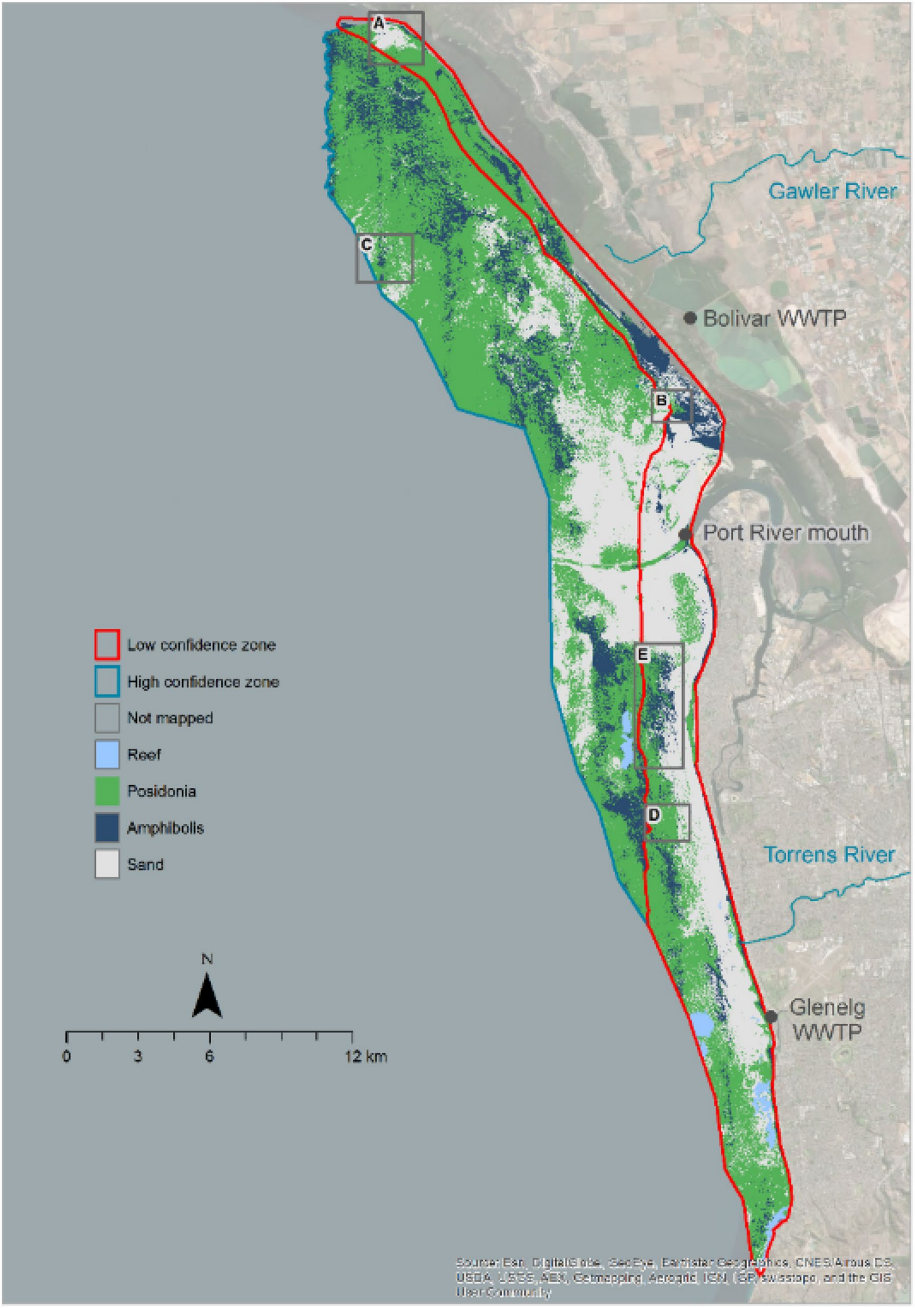
Seagrass genus classification in 2018, with annotations A–E highlighting areas discussed in the text. Map created in ESRI ArcGIS 10.7; terrestrial ESRI Basemap World Imagery (https://www.esri.com/en-us/arcgis/products/arcgis-enterprise).

Spectral differences between genera were further compromised by slight sensor saturation nearshore, leading to a ‘low confidence zone’ in classification (Fig. 3). In this zone, original spatial patterns (e.g. annotation B in Fig. 3) and visibly discernible differences between classes (e.g. annotation D) were sometimes lost. The addition of a depth variable tended to degrade classification, while the inclusion of latitude and longitude improved class discrimination, particularly in deeper regions where it prevented over-classification of *Amphibolis*. Other challenges encountered in the genus classification included the presence of suspended sediment nearshore leading to sand over-classification (annotation A), and variation of image quality between adjoining flight lines resulting in unsightly abrupt changes in classification (e.g. annotation C).

Despite limitations, overall accuracy of the genus classification was high at 85%, ranging from 80% in the low confidence zone to 93% in the high confidence zone, with only a few *Amphibolis* sites classified as *Posidonia*, and one sand site classified as *Amphibolis* (Table 2). The Cohen’s kappa coefficient of 0.76 further confirmed strong agreement between field and mapped cover, varying between 0.69 in the low confidence zone to 0.86 in the high confidence zone. The classification indicated dominance of *Posidonia* (53%) over sand (32%) or *Amphibolis* (15%) (Fig. 3). *Amphibolis* was more prevalent at the landward edge of the seagrass distribution and at mid-depths (~ 3–7 m in the north and 5–9 m in the south), but largely disappeared in a 5 km radius from the Port River mouth, and off the Gawler and Torrens rivers.

**Table 2:** Confusion matrix based on number of sites, and accuracy for the genus level classification.

Mapped cover	Reference (field) data				User’s accuracy (%)
	*Amphibolis*	*Posidonia*	Sand	Total	
**Study area**					
*Amphibolis*	**10**	**0**	**1**	11	91
*Posidonia*	**4**	**14**	**0**	18	78
Sand	**0**	**0**	**5**	5	100
Total	14	14	6	34	
Producer’s accuracy (%)	71	100	83		
Overall accuracy (%)	85				
**High confidence zone**					
*Amphibolis*	**7**	**0**	**0**	7	100
*Posidonia*	**1**	**6**	**0**	7	86
Sand	**0**	**0**	**0**	0	N/A
Total	8	6	0	14	
Producer’s accuracy (%)	87	100	N/A		
Overall accuracy (%)	93				
**Low confidence zone**					
*Amphibolis*	**3**	**0**	**1**	4	75
*Posidonia*	**3**	**8**	**0**	11	73
Sand	**0**	**0**	**5**	5	100
Total	6	8	6	20	
Producer’s accuracy (%)	50	100	83		
Overall accuracy (%)	80				

## Discussion

The study presented here is arguably the most successful mapping of benthic exposure for the Adelaide metropolitan coastline, covering a larger extent than previous studies^21–24^, and with high accuracy. The benthic cover mapping (sand *vs* seagrass) had very high accuracy (98% overall accuracy, Kappa = 0.93), and the genus classification had high accuracy (85% overall accuracy, Kappa = 0.76). These accuracies compare favourably with other similar studies, typically ranging between 50 and 75%^17,25^. The mapping success is explained by the high spectral resolution of the sensor in the range where water transparency is highest, combined with its high signal to noise and high dynamic range, further improved by pre-processing to remove sunglint^26^ and normalize illumination with depth^25^. Mapped seagrass cover however was conservative for both mapping products (benthic cover and genus) because the image spatial resolution (2 m) and classification based on similarity to either sand or dense seagrass spectral signatures hinders the detection of cm-scale patches of early colonization if these patches are not the dominant class in a pixel. The mapping approach needs further tuning to the specific mapping goal, requiring slightly different solutions for the benthic cover and the genus mapping. For example, tuning image acquisition for genus classification requires careful consideration of signal integration time to allow genus discrimination in deeper waters while minimising sensor signal saturation in the nearshore.

The benthic cover mapping revealed extensive seagrass meadows covering 70% of the study area. The genus classification suggests that *Posidonia* and *Amphibolis* distribution is intertwined, with *Amphibolis* preferentially found at the landward edge of meadows and at mid-depths. The map of genus cover corroborates observations of an *Amphibolis* province around Point Malcolm (annotation E, Fig. 3) recorded more than a decade earlier using multispectral satellite imagery^27^, and video transects, as well as the historical reconstruction of records going back to the 1970s^28^, suggesting these are long-term features of the Adelaide coast rather than a point in time in the successional sequence. Although *Amphibolis* is considered an early colonizer in highly dynamic regions through the development of deep vertical roots and wiry stems^29^, it can also become the climax species in areas with high rates of physical disturbance^15^.

The spatial pattern of *Amphibolis* distribution was disrupted in the vicinity of the Port River shipping channel, off the Gawler River, and nearshore north and south of the Torrens River, areas where existing seagrass was largely classified as *Posidonia*. Previous studies^28,30^ have raised selective loss as a mechanism to explain *Amphibolis* absence in a number of locations along the Adelaide coast affected by historically large nutrients and suspended solids inputs. The genus has a low tolerance to reduced light conditions, particularly when driven by epiphyte cover^31,32^, and its recovery in disturbed systems is considered notoriously difficult, even when loss is driven by factors other than water quality^33^. *Posidonia* instead was observed in some areas largely classified as bare sand for the last 2–3 decades^21–24,34^, e.g. the western boundary offshore of the *Amphibolis* province off Point Malcolm, and in the nearshore embayment south of the Port River mouth. Video transects (Fig. 1a) confirm the presence of *Posidonia* but mixed with *Zostera* and occasionally sparse *Halophila* and *Pinna bicolor* (razorfish). This potential recovering trend follows the decommissioning of sludge outfalls in 1993^34,35^, the closure of a soda ash factory in the Port River in 2013^36^ and continuous improvement of wastewater discharges^37,38^.

In conclusion, the use of hyperspectral imagery provides improved monitoring of seagrass dynamics at the scale of the seascape, achieving high spatial resolution and accuracy in the mapping of both benthic cover as well as genus distribution. Where inputs from land had been historically high, *Amphibolis* is absent, but *Posidonia* is still recorded and is likely recovering following a reduction in discharges. The data from this study provides a baseline from which our understanding of the responses of individual genera can be further refined in the future with subsequent mapping. Future mapping should also consider the inclusion of genera which although comparatively rare in the seascape (e.g. *Zostera*) might play a critical successional role.

## Methods

### Study area

The study area covered 784 km^2^ in coastal waters up to 17 m depth off Adelaide, South Australia (Fig. 1a). The climate is Mediterranean and rainfall occurs primarily in winter^38^. These oligotrophic waters support vast meadows of *Posidonia sinuosa*, *Posidonia angustifolia* and *Amphibolis antarctica*^28^. The physical setting is determined by prevailing waves from the southwest, generated by wind and oceanic swell^39^. The chemical setting is currently driven by discharges from three wastewater treatment plants and three rivers, all in close proximity to each other^38^. These inputs affect the light climate through suspended solids and phytoplankton in the water column, and epiphytes growing on seagrass leaves^40^. Seagrass loss was first recorded from the 1950s, peaking in the 1970s^21–24^. The total area of loss was estimated at over 6,200 ha by the late 2000s^19^. The first signs of recovery started to appear in 2013^24^.

### Field data

Nineteen sites were surveyed between March and May 2017 through the regular monitoring program of the South Australian Environment Protection Authority (EPA). Sites were selected between 2 and 15 m water depth by random sampling design using a 500 × 500 m grid overlayed on bathymetry, with the number of sites and replication within sites (transects) based on the power afforded by results from previous surveys^41^. At each 20 ha site, 10 random 50 m video transects were surveyed. Transects were undertaken using a geo-referenced 450-line analogue video camera (Scielex) angled at 90 degrees to the seafloor. A live video feed to a surface screen viewed by a trained operator ran directly from the camera into an audio and video encoding system (Geostamp) which overlays a GPS location, direction, speed, date and time strings to the video and on a hard drive. The set-up of the camera provided a field of view of approximately 1 m^2^, allowing post-processing to classify seagrass cover as either low (< 50%), medium (50–75%) or dense (> 75%) over 1 m increments. Locations used for accuracy assessment of image classification (Fig. 1a) were the centre point of a transect segment covering an area of homogenous dense cover at least 26 m long. Locations with smaller spatial extents of dense cover were used for image interpretation or definition of training areas. Field data spatial uncertainty was conservatively estimated as 12 m based on GPS uncertainty (< 10 m), and variable distance of camera from GPS (< 2 m). The EPA transects were complemented by field surveys in 2018 to locate additional areas for accuracy assessment of image classification, and to calibrate the classification in areas of suspected low cover (< 50%) in 21 additional sites of 4 transects in the vicinity of the Port River shipping channel (Fig. 1a).

### Airborne imagery

Airborne imagery was acquired in late summer (8–9 March 2018). A Diamond HK-36 ECO-Dimona aircraft was fitted with a hyperspectral linescanner (modified Specim AISA Eagle 2) acquiring 62 spectral bands of 9.5 nm from 408.5 to 990.6 nm. The raw data was recorded with GNSS time for the centre of the integration period for each captured frame. The spatial resolution was 2 m, and integration time 25 ms, an interval selected to just reach sensor saturation on land and maximise the ability to image deeper waters. North–South flight lines were oriented as near as practical to the direction of the solar azimuth to minimize cross-track bidirectional reflectance and sunglint. The image swath was approximately 1900 m, with 1 km spacing between tracks to allow for overlap. Supporting instrumentation included an RT-4003 precision navigation unit (Oxford Technical Solutions) incorporating a dual-GNSS system and IMU rigidly mounted with the hyperspectral sensor.

Post-processing combined the raw aircraft GNSS and IMU data with data from a deployed NovAtel DL-V3 GNSS ground station as well as publicly available base stations and post-hoc precision satellite ephemeris data. Accurate frame timing allowed correlation with post-processed navigation data to deliver location and 3-axis orientation of the optical axis for each frame. Imagery spatial uncertainty was conservatively estimated as 8 m based on IMU position and orientation performance, instrument optics and timing accuracy of the hyperspectral frames over the integration time. Raw hyperspectral frames were processed to at-sensor radiances using a 2015 radiometric calibration, with five seconds of dark-frame data collected at the end of each flight line. ATCOR-4 software^42^ was used to derive at-surface reflectances, assuming a standard library maritime atmosphere with 70 km visibility.

### Imagery classification

Image processing and map production was performed across Python, ENVI and ArcGIS. Sunglint removal and depth normalisation algorithms were implemented in Python. Spectral subsetting, imagery classification, majority filtering, and J-M distance were all performed in ENVI 5.3. Maps were produced in ArcGIS 10.7.

#### Benthic cover classification

Imagery pre-processing involved sunglint removal^26^ and spectral subsetting to exclude bands with poor signal-to-noise ratio interpreted as substantial visible random variation in brightness within cover types. Bands 7 (463.0 nm) through to 21 (593.6 nm), and band 62 (990.6 nm) were retained. After pre-processing, training areas of 5 × 5 pixels were defined for each substrate class in each flight line until approximately 2,000 pixels were selected based on visual interpretation and field data. Training areas of 5 × 5 pixels encompassed an area large enough (100 m^2^) to locate with confidence given spatial uncertainty (< 12 m), and using 2,000 pixels identified 80 distinct training areas, a number that represents a good balance between optimising training time and selecting enough appropriate training areas in the available field data. The bare substrate class is referred to as sand, and the non-substrate class as seagrass. Explicit distinction of macroalgae was not possible due to the absence of known macroalgae areas for classification training, but macroalgae cover is expected to be small. The location of reefs was supplied as shapefiles by the Department for Environment and Water of South Australia.

A Mahalanobis distance supervised classification was applied to each flight line, and classified imagery mosaicked into a single map. In areas of overlap priority was given to higher quality imagery, i.e. where wave action was lowest, or where turbidity was lowest, or where sunglint was minimal, or in areas of sunglint, where sunglint removal was more successful. The Mahalanobis classifier was chosen as the most successful after testing several classifiers. As training areas had medium to dense seagrass, pixels classified as seagrass are estimated to contain > 50% seagrass cover. Majority filtering (10 × 10 m) was used to reduce occasional pixel misclassification related to wave action, turbidity and sunglint. Terrestrial and intertidal areas were masked, as well as all areas of low- or no-confidence due to water depth, cloud cover or shadow, or suspended sediment, as well as areas of seagrass wrack and breaking waves on beaches.

#### Genus classification

Genus classification included spectral subsetting and sunglint removal as described above, as well as illumination difference normalisation with depth^25^. Training areas of 5 × 5 pixels were defined for each substrate class in each flight line based on visual interpretation and field data until approximately 1500–3000 pixels were selected (more for flight lines with more within- and between-class variability). The image was classified into *Amphibolis*, *Posidonia* and sand, using Support Vector Machine (SVM) supervised classification with linear function kernels. The SVM classifier was chosen as the most successful after testing several classifiers. The effect of including depth and latitude/longitude variables in the supervised classification was also tested. The mosaicking of classified flight lines into a single map of genus cover included majority filtering (10 × 10 m) and masking. In addition, the spectral separability of *Amphibolis* and *Posidonia* with bathymetric depth was calculated by J-M distance for training spectra recorded at field data locations, with 0 indicating no separability and 2 complete separation. The bathymetry^40^ was derived from the Adelaide Coastal Waters Study^39^ in the Port Adelaide and Barker Inlet area, and from the Australian bathymetry and topography grid^43^ elsewhere. Reference sites of approximately 5 × 5 pixels were selected for both seagrass genera from 1 to 11 m water depth, including 9386 *Amphibolis* pixels and 14,025 *Posidonia* pixels.

#### Accuracy assessment

The combined 2017/2018 field dataset allowed accuracy assessment of the sand (n = 10) versus seagrass classification (n = 30), as well as accuracy assessment of the genus classification into *Amphibolis* (n = 14), *Posidonia* (n = 14), and sand (n = 6). Standard spatial accuracy statistics were computed, including Overall accuracy (% of reference sites mapped correctly), Producer’s accuracy (% of real features mapped correctly), and User’s accuracy (% of classes mapped correctly). The Cohen’s kappa coefficient was also calculated, with a value of 0 suggesting chance agreement between field and mapped data, and 1 complete agreement.

## Data Availability

The datasets generated during the current study are available from Figshare on 10.25909/14752005.
