# Author Correction: Using hyperspectral imagery to investigate large-scale seagrass cover and genus distribution in a temperate coast

**DOI:** 10.1038/s41598-021-96544-9

**Published:** 2021-08-19

**Authors:** Kenneth Clarke, Andrew Hennessy, Andrew McGrath, Robert Daly, Sam Gaylard, Alison Turner, James Cameron, Megan Lewis, Milena B. Fernandes

**Affiliations:** 1 School of Biological Sciences, The University of Adelaide, Adelaide, SA 5005 Australia; 2 Airborne Research Australia, PO Box 335, Salisbury South, SA 5106 Australia; 3 SA Water, GPO Box 1751, Adelaide, SA 5001 Australia; 4 South Australian Environment Protection Authority, GPO Box 2607, Adelaide, SA 5001 Australia; 5 Department for Environment and Water, GPO Box 1047, Adelaide, SA 5001 Australia; 6 College of Science and Engineering, Flinders University, GPO Box 2100, Adelaide, SA 5001 Australia

Correction to: *Scientific Reports* https://doi.org/10.1038/s41598-021-83728-6, published online 18 February 2021

The Data Availability statement in the original version of this Article was incorrect.

“The datasets generated during the current study are available from the corresponding author on reasonable request. Once the paper is published, these datasets will be submitted to Dryad.”

now reads:

“The datasets generated during the current study are available from Figshare on https://doi.org/10.25909/14752005.”

The original Article has been corrected.

